# Genetic research on rare familial disorders: consent and the blurred boundaries between clinical service and research

**DOI:** 10.1136/jme.2006.018564

**Published:** 2008-08-22

**Authors:** M Ponder, H Statham, N Hallowell, J A Moon, M Richards, F L Raymond

**Affiliations:** 1 Centre for Family Research, University of Cambridge, Cambridge, UK; 2 Public Health Sciences, University of Edinburgh, Edinburgh, UK; 3 Department of Medical Genetics, Cambridge Institute for Medical Research, Addenbrookes Hospital, Cambridge, UK

**Keywords:** documented consent, research ethics, genetic research, rare diseases

## Abstract

**Objectives:**

To study the consent process experienced by participants who are enrolled in a molecular genetic research study that aims to find new genetic mutations responsible for an apparently inherited disorder.

**Design:**

Semi-structured interviews and analysis/description of main themes.

**Participants:**

78 members of 52 families who had been recruited to a molecular genetic study.

**Results:**

People were well informed about the goals, risks and benefits of the genetic research study but could not remember the consent process. They had mostly been recruited to take part by trusted clinicians or their relatives but had little memory of, or concern about signing consent forms. Families appeared to regard the research as a continuation of their, or their relatives’, clinical care.

**Conclusions:**

Ethical review should be more flexible in its attitude to consent forms and written information sheets for some sorts of research. For rare genetic disease studies where research has been discussed fully within the clinical setting then the consent obtained at that time could suffice rather than needing extra consent at a later stage. However, clinician-researchers will need to ensure that their duty of care extends for the duration of the research and beyond.

The Declaration of Helsinki underpins the conduct of research with human subjects.^1^ It sets out clearly the responsibilities of clinicians and researchers to make sure that participants are not subjected to undue risk of harm and it puts informed consent as a fundamental requirement.^1^ Ensuring that written records of consent are kept is perceived as essential, for example, see the UK Human Tissue Act^2^ and UK Biobank^3^. Some researchers have raised concerns that insisting on written consent may impede certain types of research.^4^

The UK National Research Ethics Service^5^ has clear guidelines for the design and content of information sheets and accompanying consent forms. The effect is to force researchers to follow a consistent pattern despite variability in the type of research being undertaken. This emphasis on the documentation of consent may lead medical researchers towards functional consent,^6^ whereby the responsibility to understand and acknowledge any risks that may be incurred by participation in research are transferred from researchers to participants. This should enhance research participants’ autonomy (p308)^7^ but how much they understand the information sheets is hard to assess,^8^ as is the degree to which consent is based on the trust relationships^9^ which exists between the researcher and participants or the context in which they are being asked to participate (pp313–4).^7^

Consent for genetic research is seen as particularly sensitive because it may have consequences for family members beyond the individual. Indeed, the research may only be possible if multiple members of the same family participate.^10^ To illustrate, once an individual is identified as potentially carrying a genetic disorder, the proband and immediate family will be asked to provide a family tree identifying those people within the family affected by the disorder. Selected family members will be approached and asked for information and sometimes blood samples for genetic analysis. Issues relating to consent and the possible coercion of relatives to take part need to be handled sensitively by researchers. Similar ethical dilemmas may arise in the clinical setting.^10^ ^11^ In the UK, clear guidance for how consent should be sought for genetic testing within clinical genetics practice has recently been published which takes account of all these difficulties.^12^

In the clinical setting, if known disease-causing genetic mutations are found, then individuals within the family can be approached via their relatives and offered the opportunity to see whether or not they carry the mutation. Appropriate genetic counselling before gene testing will usually be offered. If no known genetic mutation is identified in the routine genetics clinic, then sometimes families are asked if they wish to extend the investigation further. As further testing is in pursuit of new knowledge and for the benefit of a wider population of people than just the family concerned, the process becomes a research activity. There may not be definite outcomes or any clear time scale, there may be uncertainties about who ultimately is responsible for conveying research findings to participants and from whom they will get clinical care.^13^ At this stage the rules governing research, (eg, participant information sheets and consent forms to be signed) take over, even though in the eyes of the family the process may seem unaltered from what has happened before. Little is known about how these families perceive research activity which appears as a continuation of their clinical care.

A molecular genetics research project is underway that allows us to explore some of these issues. The Genetics of Learning Disabilities (GOLD) Study aims to find new mutations in new genes that may be responsible for intellectual disability (ID) in families with two or more affected males where the cause of the ID is unknown despite full investigation. This international study has recruited several hundred families where there are two or more males with ID.^14^ The assessment of ID varied depending on the referral route: some were totally dependent for all their needs whilst others could live relatively independently. The data upon which this paper is based derives from a separate, but linked, study whose aim was to determine the expectations and experiences of GOLD study participants resident in the UK or Ireland—the Family study.

This paper focuses upon participants’ experiences of the initial consent process for the GOLD study. It describes how these families came to participate, their experiences of giving consent and their knowledge, expectation and understanding of the research to which they have consented. These data will be used to comment on the operating framework for research on rare genetic disorders in the UK. Although in the GOLD study the genetic disorder under investigation is ID, this paper does not attempt to discuss the issues relating to consent for participating in research from those family members with reduced or lack of mental capacity. This is a complicated issue which raises a number of practical and ethical problems and will be addressed fully in a separate paper.

## METHODS

UK families were recruited to the GOLD study through their local clinical geneticist and most were seen either by LR or JM in their homes. Blood samples were taken from individuals for molecular genetic analysis. Individuals included affected males, their mothers, brothers, unaffected male cousins and their mothers (aunts of affected males). Occasionally, family members were not seen in person by the GOLD study team but were given written information and asked to have blood samples taken locally. All GOLD study participants were given an information sheet which stated that: the study was research to find the genetic causes of ID; blood samples would be needed; results, if any, may take several years; who the researchers were; and that there was no obligation to take part. They were asked to sign consent forms and proxy consent was requested for children and those who lacked capacity to do it themselves.^i^

Members of 90 GOLD study kinships from the UK and Ireland were invited to participate in the Family Study, which was reviewed and given a favourable opinion by the London multi-centre Research Ethics Committee. Initially, invitations were sent to selected families who had already become part of the GOLD study and where preliminary genetic research analysis showed they were the most likely ones to have a gene mutation found. Subsequently all families who joined the GOLD study were visited by the research nurse and all of these families were invited to participate in the Family Study.

For the Family Study, two methods of recruitment were employed—a direct approach from the GOLD study team, and secondary recruitment by one family member to another after the initial contact. Those recruited directly were either sent our information packages by the clinical geneticist or given them by the research nurse (JM) when visiting a family. These packages comprised an information sheet, consent form and pre-paid reply envelope. Individuals were asked to return consent forms to the researchers in Cambridge. Some returned the consent form, but others indicated that they would prefer to be contacted directly by telephone. Respondents were contacted by HS or MP to arrange an interview. We could only access relatives through the person we first interviewed. Prior to interview most had signed consent forms and at interview consent was reconfirmed.

All interviews were tape recorded and transcribed verbatim. Organisation of the data for analysis was aided by the use of ATLAS-ti.^15^ A thematic analysis was undertaken in which recurring themes, within and across interviews, were identified.^16^ HS and MP crosschecked transcripts to verify emergent themes. The interview followed a schedule with prompts and was developed by MP and HS during a period of consultation and pilot interviews with families affected by Fragile X syndrome. The topics covered ranged from factual information about their families, their experiences of having ID in family members, and their experience, knowledge, expectations of and understanding of the GOLD study. This paper focuses upon a subset of the data which concentrated on participants’ experiences of becoming involved in the GOLD study and their memory and understanding of the consent process.

## RESULTS

### Characteristics of the study participants in the family study

Members of 35 kinships (39% of 90 invited) took part in this study. Overall, 114 individuals from 52 families (some kinships comprised several families) agreed to participate. Eighty-four individuals had either donated a blood sample to the GOLD study or been part of the consent process for an affected person. The remaining 30 relatives had not been required to give a blood sample for the GOLD study and so the data from their interviews is not included in this analysis. This paper reports data from 78 participants: data from six interviews with those with ID will be reported elsewhere.

### Remembering recruitment to the GOLD study

Our participants reported that they came to participate in the GOLD study because they wanted to find the cause of the ID in their family. So how did they experience the recruitment process? Many of those who had given blood or proxy permission for their son/brother talked about how they had been asked to participate. Like the respondent below, most had been approached by a known healthcare professional as a continuation of either their own, or a relative’s, clinical genetics consultation.

I: And then how did you come to be recruited …

R1: Professor [geneticist], he suggested it, he said that they couldn’t find any results, because they checked [son] for fragile X and obviously the Downs bit ... but there was nothing in that … which was good, all the results came back fine, but he said that there was somewhere in Cambridge, and he explained about [research nurse] and what she does, and would I be interested. And I said yeah, I wouldn’t mind doing that, and then he just explained well … if you don’t want it done it doesn’t mean we’re going to cross you off our books and … and I said well no, that’s fine, and then it was left for quite a while, and then I was written to and I thought yeah, I’ll do this then. (GO_22_01 mother and sister)

Some were contacted months/years after attending a clinic. These individuals said they were pleased to receive the invitation to participate “out of the blue” because they felt that this was an indication that something was being done about the situation in their family:

I: So this information about the GOLD Study came out of the blue. Can you talk me through what happened, you know, sort of how you heard, what went on?

M: [research nurse] contacted us first to say she’d be interested in doing blood tests.

I: Right, and that was the first you’d heard?

M: Yes, after Dr [paediatrician] sent off the letters. And we were just delighted that some sort of investigation … because it’s too bland to say there may be a genetic link, I wanted proof there was a genetic link. (GO_15_01 mother, last contact with genetics service 3–4 years previously at her instigation)

Several families had participated in previous genetic research studies and therefore, had given blood samples before, sometimes many years ago. These families were also pleased to be approached to take part in the GOLD study:

I: How did you get recruited to that (GOLD) can you remember?

R: Through Dr [geneticist seen 12 years previously], he phoned me up and I thought I’d never hear from him again. So that was quite a surprise.

I: So you had had blood samples taken when [son] was about 9 months and this was the next time since then? How did you feel about being phoned up?

R: You know I was quite pleased really because it was results and then even better when he said there was someone in Cambridge who was interested in coming up to see us and finding out what sort of help we could get—I thought it was really good because I had really given up hope. (GO_10_01 mother and sister)

These three responses illustrate how the majority of participants remembered the initial approach. Many could name the clinician who had approached them because often s/he was responsible for their routine care. A minority of our participants could not recall who had made the initial approach for the GOLD study.

Members of the extended family, who had not been to a clinic, recalled how a relative had approached them with information about the study and a request to participate:

I: So how did it come that you were all involved in this GOLD Study of the blood samples, can you remember how all that came about?

R1: Oh, [cousin] … most of this has come from [cousin].

I: That’s all come from [cousin]? Right, and can you talk me through what happened, who told who what …

R1: Well I don’t know, [cousin’s mother] … there was talk of something going on, but it didn’t actually come to anything. But we said well, if there is anything going on, we’ll obviously be involved. (GO_03_05 grandmother)

Thus, the data suggest that our participants were easily able to recall the initial invitation to participate.

### Knowing the goals, risks and benefits of the research

To be informed about research, participants need to know what the research is about, what will happen to them, and what are the potential harms and benefits.^3^ The MREC-approved information sheet for the GOLD study covered all these topics.^i^ Potential study participants were informed that: faulty genes sometimes cause learning disability; blood samples would be taken; a result may take up to five years, but not all families could expect to gain a result; and, where a result was found, it would help provide answers to the questions “Why does our child have learning disability?” and “Will our next child have learning disability?” They were also told about issues of confidentiality, about their right to decide to take part and withdraw.

All the people we interviewed were aware that the aim of the GOLD study was to look for causes of ID in families. Most knew it was genetic research, and all had a realistic expectation that the results, if any became available, were likely to take a very long time in coming:

R: Oh well they sent me a letter about the genetics part of it and said could she come down and take blood samples, and she came down and she took blood samples and more or less said that there was other families involved and it could take about five years. (GO_05_01 mother and sister)

Although a few people thought a “diagnosis” would help themselves, or other family members, to access care or benefits, most knew the research was unlikely to help those currently affected with ID, but perceived the research as potentially helping people in the future.

... of course, I mean if it’s going to benefit people in years to come. Like there’s no benefit for us apart from the knowledge that… if they can find something out (GO_15_03 mother)

All but one knew that genetic research may lead to a test which in turn could lead to more informed reproductive choices, (the ID in this participant’s family was mild). This aspect of research participation was very important to those of our respondents who wanted to prevent future boys with ID being born into their family:

R: Well the main reason we’ve taken part is… for my daughter and my sister’s two daughters… So that perhaps they can be screened before having any children (GO_O7_01 mother, sister and aunt)

Potential risks included unrealistic expectations about what might be achieved or how quickly and the potential for disappointment if no results emerged. However, our study participants appeared to understand these risks. Many talked about the time lag between giving blood and gaining a result, and the possibility that tangible results may not emerge from the research:

M: … well, it’s a four year study I think, and … well I think they would contact us certainly if they could find anything specific, if they don’t, I’m not sure whether to expect to hear from them or not. But it was very informative at the time of giving blood, the person who came. (GO_03_03 mother)

Thus it would appear that many of those interviewed were aware of the goals of the GOLD study and the potential risks and benefits to their family and others. In this sense at least, their consent can be regarded as informed. However, as the following section demonstrates few could recall the details of the consent process, such as signing a consent form.

### Recalling formal aspects of the consent process

All participants of the GOLD study were given a five-part consent form to sign along with the information sheet. The form specified consent to take part, withdraw without notice, access to medical records, a blood sample being taken and the establishment of cell lines. These were signed by participants or their proxies. However, many appeared unable to recall signing the form:

R: I signed a form when I was with [researcher], but I can’t remember what it was for. (GO_04_01 mother and grandmother)

I: Did you sign anything? Any consent forms, do you remember?

R: I might have done, I can’t remember. (GO_07_01 mother who gave a particularly good account of what the research was about)

This may be explained by three factors. First, participants appeared to find the decision to participate in the study uncontentious:

I: So was it at all difficult to decide to take part or was it something you had to talk about among yourselves or …?

R1: No, no it wasn’t difficult was it, it was just something … I think I made the decision to go, look into it yeah. (GO_18_04 mother and aunt)

I: Did you feel there was any sort of decision needed to be made about whether or not you wanted to take part?

R: No, no question. Because unless we support you, how can you support us? (GO_07_01 mother)

Secondly, they said that once they had given blood they had rarely thought about the study beyond sometimes wondering if there would be a result:

I: Is it something you think about?

R: Well yes, obviously you think about it from time to time, and just wonder where it’s going, or is there ever going to be any answers, that’s what I always think, is there going to be any. I’m not saying it’s a waste of time, I’m just saying what’s at the end of the road, is there anything there? (GO_06_01 mother and grandmother)

And thirdly, as illustrated above, they had remembered the telephone calls, letters and consultations where the GOLD study had first been introduced as the point or time at which they had agreed to take part. Thus the subsequent receipt of the Patient Information Sheet (PIS) and signing the consent form appeared less memorable.

Similarly, those recruited through relatives had little recollection of the formal consent procedures, such as receiving the PIS or signing a consent form:

I: Did you have to sign a consent form before you gave blood, were you given any written information?

R1: [long pause]

R2: No, I’d have seen it wouldn’t I.

R1: Well you weren’t there [husband], were you?

R2: No, but you’d have brought it back, if you’d had anything given to you …

R1: I don’t … no, I don’t think so, no.

I: Right, so there was nothing …

R1: No, not that I can remember. (GO_03_ 05 & 08 grandparents and cousin)

It was clear that some parents of affected boys had expectations that their relatives would participate in the GOLD study by providing blood samples. As the quotation below illustrates, they felt their relatives had a duty to do this even if those family obligations did not extend to helping with practical care of people with ID:

*…* The rest of the family, I insisted that they have the blood test. I don’t care whether they support me with the children or this or that, but … I think they are duty bound to provide a blood sample. And that’s all that’s expected of them. I don’t want any more, just the blood samples. (GO_15_01 mother)

We do not know what this woman’s relatives would say about their memory of consent nor to what extent they might have felt coerced to participate. However, a number of participants from other families told us that they had willingly taken part in the GOLD study because they wanted to help their family:

I: How were you told about the study? Did it come from the doctors or from [daughter]?

R: It’s all come from [daughter].

I: It all came from her. And she roped you into—sticking your arm out …

R: I don’t mind. It was really, she just said, oh well such-and-such got in touch with me and they want your blood. And I said all right. That’s more or less how it’s come about. But we’re quite prepared to go along with it. (GO_06_01 mother and grandmother)

## DISCUSSION

In this paper we have reported data describing participants’ recall of how they came to take part in a research study which sought to determine the genetic basis of learning disabilities in families (the GOLD study). They recalled being recruited to the GOLD study by either a familiar clinician or members of the GOLD team, and were reasonably well informed about the goals, risks and benefits of the research. Recent legislation^2^ requires explicit consent for participation in research that uses human tissue samples, and there is a general presumption that any consent to research will be documented.^5^ Our study suggests that although participants recalled agreeing to participate and receiving information about the research from their clinician/study team or family members, the formal process of giving signed consent was less memorable. Although we cannot be sure that the written consent process had not helped to reinforce the accuracy with which participants appeared to recall the GOLD study, these observations do raise questions about the role and purpose of written consent in research.

One explanation for our participants’ lack of recall of providing written consent and receiving the patient information leaflet may be that they did not perceive the procedures carried out as part of the GOLD study as differing from the clinical care they or their relatives had previously received. For these families, normal clinical care would have included family history taking, blood sampling and relatives being asked to give information and blood samples, just as happened when they became part of the GOLD study. Thus, because this process was similar to what had previously happened participants may have paid less attention to the formalised procedures for obtaining their consent for research. Furthermore, because our participants had been unable to obtain a diagnosis for the ID in their family via a clinical route, they may have perceived research participation as a logical next step in their search for an answer. Clinicians in this sub-specialty regularly invite their patients to enter into research studies in order to identify disease-predisposing genetic mutations. These observations support Parker *et al*’s^17^ views that the boundary between research and clinical practice in rare genetic disorders is becoming increasingly ambiguous, for both clinicians and patients. However, the push from regulators has been to insist on a clear boundary between research and clinical activity.

The data support the arguments that consenting in research should be seen as a process rather than as a single event^7^ ^18^ and that the process should recognise the specific context of those being asked to give consent. The process of consenting to take part in the GOLD study took place for these families at a stage when they had already embarked on a process of genetic investigation. The point at which it slipped from being a purely clinical activity into a research activity was not significant to them. This does not imply that they did not know what they were doing or were uninformed, merely that these families did not see an important distinction between what was research and what was not.^17^ Clinical care evolved towards research and families reported that they felt adequately informed along the path. The challenge for researchers is to ensure that participants continue to be informed and are adequately cared for throughout the research and after it ends.

It must be noted that there are limitations to this study. The families who took part in our study are a select group. They are all participants in a molecular genetics study (the GOLD study) and data about those who declined to participate are unavailable. Of those who participated and were invited to join this additional family study, only 40% agreed. We know nothing about those individuals who either declined our invitation, or who were not invited by their relatives. Our families have a very favourable view of research because they appear to perceive it as their only route to getting a diagnosis which may help their families in the future. The condition being investigated in the GOLD study was ID and we cannot assume that those who participate in genetic research for other types of disorder will report similar experiences.

## CONCLUSION

Research has become regulated in an effort to accommodate all types of research in a single process. This has led to the formalisation of consent procedures, such as providing comprehensive information leaflets and long and complex written consent forms. These are seen as essential to protect people from harm in drug trials or other risky interventions. However, our data suggest that the current emphasis on signed consent may be less relevant in some circumstances. In an exploratory genetic study such as the GOLD study, our participants indicated that written consent was not an issue because the research was seen as a continuation of a process already started in the clinic. Moreover, in most cases, the person inviting them to participate was the clinician responsible for their care with whom they had a trusting relationship.^9^ We would always endorse that consent to participate in research is necessary and desirable. However, we believe that there could be more flexibility in the consent procedures required by ethics committees, such that they fit with the particular type of research being undertaken. Clinical genetics practice now includes documenting that people have agreed to genetic testing and that they understand all possible consequences.^12^ This agreement should suffice for research activity which is a direct continuation of clinical practice. However, this means that when investigations do move into the research phase, clinicians and clinician-researchers must ensure that they have discussed the specific research fully and that high standards of clinical care continue for their patients throughout the research period. This may need to be addressed more explicitly than is done at present through the system of annual and/or end of study reports required by ethics committees.

## Footnotes

i The consent form and information sheet for families participating in the research study of the genetics causes of learning disability (GOLD) can be accessed at: http://goldstudy.cimr.cam.ac.uk/consentforms.htm.

## References

[1] World Medical Association Declaration of Helsinki Ethical principles for medical research involving human subjects. Bull World Health Organ 2001;19:313–14 PMC2566407 11357217

[2] Human Tissue Act 2004 http://www.opsi.gov.uk/acts/acts2004/20040030.htm (accessed 10 June 2008)

[3] UK Biobank http://www.ukbiobank.ac.uk (accessed 10 June 2008)

[4] Peto J Fletcher O Gilham C Data protection, informed consent, and research. BMJ 2004;328:1029–30 15117769 10.1136/bmj.328.7447.1029 PMC403832

[5] National Research Ethics Services http://www.nres.npsa.nhs.uk/ (accessed 10 June 2008)

[6] Alderson P Goodey C Theories of consent. BMJ 1998;317:1313–5 9804727 10.1136/bmj.317.7168.1313 PMC1114211

[7] Berg JW Appelbaum PS Lidz CW Informed consent: Legal theory and clinical practice. 2nd Edn. Oxford: OUP, 2001

[8] Helgesson G Ludvigsson J Gustafsson Stolt U How to handle informed consent in longitudinal studies when participants have a limited understanding of the study J Med Ethics 2005;31:670–3 16269567 10.1136/jme.2004.009274 PMC1734044

[9] O’Neill O Some limits of informed consent J Med Ethics 2003;29:4–7 12569185 10.1136/jme.29.1.4 PMC1733683

[10] Beskow LM Botkin JR Daly M Ethical issues in identifying and recruiting participants for familial genetic research. Am J Med Genet A 2004;130:424–31 15455364 10.1002/ajmg.a.30234

[11] Parker M Lucassen AM Genetic information: a joint account? BMJ 2004;329:165–7 15258076 10.1136/bmj.329.7458.165 PMC478234

[12] Royal College of Physicians, Royal College of Pathologists, British Society for Human Genetics. Consent and confidentiality in genetic practice: guidance on genetic testing and sharing genetic information. Report of the Joint Committee on Medical Genetics. London: RCP, RCPath, BSHG, 2005

[13] Biesecker BB Peay HL Ethical issues in psychiatric genetics research: points to consider. Psychopharmacology (Berl) 2003;171:27–35 13680086 10.1007/s00213-003-1502-2

[14] Tarpey P Parnau J Blow M Mutations in the DLG3 gene cause nonsyndromic X-linked mental retardation. Am J Hum Genet 2004;75:318–24 15185169 10.1086/422703 PMC1216066

[15] Muhr T ATLAS-ti—computer aided text interpretation and theory building. Berlin: User’s manual, 1994

[16] Miles M Huberman AM Qualitative data analysis. 2nd Edn. Thousand Oaks, CA: Sage Publications, 1994

[17] Parker M Ashcroft R Wilkie AOM Ethical review of research into rare genetic disorders. 10.1136/bmj.329.7460.288. BMJ 2004;329:288–9 15284160 10.1136/bmj.329.7460.288 PMC498040

[18] Corrigan O Empty ethics: the problem with informed consent. Sociol Health Illn 2003;25:768–92 10.1046/j.1467-9566.2003.00369.x 19780205

